# The Physiology of the Carbohydrates

**Published:** 1896-09

**Authors:** 


**The Physiology of the Carbohydrates.** An Epicriticism by F. W. Pavy, M.D., F.R.S. Pp. xix., 141. London: J. & A. Churchill. 1895.

This volume gives a very interesting record of the difficulties which the founders of "new truths" have to encounter. The author is well able to defend himself, and does so with vigour, both as regards the criticism of the physiologist, and the officers of the Royal Society. He submits that Dr. Paton's criticism has nowhere touched even the fringe of his position, and it seems likely that the portals of the Philosophical Transactions will be somewhat more zealously guarded in the future.

